# Stage-dependent shifts in native and invasive traits mediate community invasibility in subtropical urban ecosystems

**DOI:** 10.1016/j.pld.2025.06.007

**Published:** 2025-06-24

**Authors:** Chengwei Li, Qi Wu, Cheng Du, Laihong Gu, Xingchen Wang, Jiajie Xie, Jiayi Wang, Jianhua Chen, Yunquan Wang

**Affiliations:** a College of Life Sciences, Zhejiang Normal University, Jinhua 321004, Zhejiang, China; b School of Life Science and Technology, Shanghai Tech University, Shanghai 201210, China; c State Key Laboratory for Vegetation Structure, Function and Construction, College of Life Sciences, Zhejiang University, Hangzhou 310058, Zhejiang, China

**Keywords:** Biological invasion dynamics, Multidimensional functional traits, Trait-mediated biotic resistance, Stage-dependent coordination strategies, Subtropical urban ecosystems

## Abstract

Biological invasions threaten biodiversity and ecosystem stability through stage-dependent functional trait mediation. However, the mechanistic linkages between invasion intensity and multidimensional functional traits remain inadequately characterized. To address this gap, we analyzed eight multidimensional functional traits across 290 subtropical herbaceous plots in Jinhua, China. By integrating invasion level, we evaluated how native and invasive species traits differentially regulate community invasibility, a metric quantifying a community’s susceptibility to biological invasion. Functional and taxonomic diversity exhibited hump-shaped patterns, peaking at moderate invasion before declining sharply under heavy invasion, while community invasibility increased markedly with invasion level. Native communities resisted invasion through persistent suppression of canopy height and stage-adaptive strategies: Leaf thickness emerged as a critical resistance trait under heavy invasion, counteracting invasive dominance. In contrast, invasive species initially prioritized rapid canopy occupation via height-mediated advantages, subsequently shifting toward stress tolerance (e.g., thickened leaves) and resource reallocation (e.g., root-shoot ratio adjustments) to consolidate dominance. Native abundance universally suppressed invasibility across all invasion stages, whereas invasive abundance amplified success only at advanced stages. Resistance was governed by stage-dependent trait trade-offs: Native leaf dry weight enhanced invasibility under light invasion but became ineffective as competition intensified. Conversely, invasive aboveground biomass and root-shoot ratio consistently promoted invasibility, reflecting prioritization of rapid resource acquisition. Our findings demonstrate that invasion outcomes depend on the spatiotemporal coordination of multidimensional functional traits. We propose an adaptive management framework for urban ecosystems emphasizing structural preservation (e.g., maintaining native canopy height) combined with stage-specific trait optimization of resistance traits to mitigate invasibility.

## Highlights

• Functional and taxonomic diversity showed hump-shaped patterns, while community invasibility increased with invasion level.
• Native communities resisted invasion by consistently suppressing canopy height and stage-adaptive strategies.
• Invasive species shift from rapid colonization traits to stress-tolerant strategies as invasion intensity increased.
• Plant invasion outcomes depend on spatiotemporal coordination of multidimensional functional traits.

## 1 Introduction

Biological invasions pose escalating threats to global biodiversity and ecosystem functioning, driven the synergistic effects of anthropogenic disturbances and global climate change (Livingstone et al., 2020; Angulo et al., 2021; Gioria et al., 2023). Elton’s biotic resistance hypothesis remains central to invasion ecology, proposing that species-rich communities resist invaders more effectively than species-poor systems (Elton, 1958; Cheng et al., 2024; Larsen et al., 2024; Hu et al., 2025). Although foundational for invasion management, this hypothesis exhibits context-dependency, modulated by three key factors: Spatial scales, invasion stages, and community assembly processes (Bach et al., 2022; Li et al., 2022b; Guo et al., 2024a; Liu et al., 2024b). Crucially, invasion success hinges on dynamic interactions between native functional traits and invader strategies throughout establishment phases (Richardson and Pyšek, 2012; Delavaux et al., 2023). Despite progress, the stage-specific mechanisms underlying biotic resistance remain poorly understood, particularly in anthropogenically stressed urban ecosystems where trait-mediated competition drives invasion outcomes (Guo et al., 2024a; Liu et al., 2024b; Yang et al., 2024).

Biotic resistance and invasion success are mediated by both direct resource competition (e.g., light interception) and indirect apparent competition (e.g., herbivory) between native and invasive species (Wang et al., 2018; Cui et al., 2023; Zhu et al., 2023). These interactions are primarily driven by multidimensional functional traits that regulate resource acquisition, allocation, and survival strategies (Gross et al., 2013; Li et al., 2015; Zheng et al., 2018; Liao et al., 2021; Liu et al., 2023). Canopy structure traits such as plant height critically determines light competition hierarchies during early invasion stages (Wang et al., 2021), while simultaneously shaping habitat suitability for subsequent establishment (Wang and Wan, 2021). Leaf traits, including leaf thickness (LT) and leaf area (LA), mediate trade-offs between rapid colonization (fast-return strategies) and persistent competition (slow-return strategies) through growth-herbivory resistance trade-offs (Reich, 2014; Catford et al., 2019; Palma et al., 2021; Fridley et al., 2022; Liu et al., 2022; Zhao et al., 2024). Biomass allocation strategies reflect trade-offs between aboveground dominance and soil resource acquisition (Wang et al., 2014; Rathee et al., 2021; Zhu et al., 2023), whereas allometric traits (e.g., specific leaf area, SLA; root-shoot ratio, RSR) optimize resource use efficiency under invasion-induced stress (Godoy et al., 2012; Wang and Wan, 2021; Larsen et al., 2024). Collectively, these trait syndromes determine whether communities can effectively resist invasion or ultimately succumb to invader dominance (Violle et al., 2007; Liu et al., 2024a).

Native and invasive species exhibit fundamentally distinct trait strategies that shape opposing invasibility dynamics. Native communities employ specific trait combinations to intensify resource competition (Emery and Gross, 2007). For instance, canopy height dominance suppresses invaders’ light acquisition (Gross et al., 2007; Thomson et al., 2011; Wang et al., 2019), while thickened leaves enhance herbivore resistance and stress tolerance (Zhao et al., 2024; Wang et al., 2025). Belowground traits further enhance resistance by promoting preferential root allocation, which establishes localized resource competition barriers (Zhang and van Kleunen, 2019). In contrast, invasive species utilize distinct functional traits and strategies to gain competitive advantages across invasion stages (Guo et al., 2015; Su et al., 2023). Early-stage height advantages facilitate reproductive success and dispersal (Wang and Wan, 2021), whereas plastic biomass allocation (e.g., RSR adjustments) sustains their dominance under competition (Zhu et al., 2023). Consequently, elucidating how native and invasive traits differentially regulate community invasibility provides critical insights for predicting invasion trajectories and designing targeted management strategies.

Biological invasions progress along the introduction-naturalization-invasion continuum, exhibiting stage-dependent dynamics (Guo et al., 2022, 2024b; Qian, 2023). During early establishment phases (e.g., introduction and naturalization), abiotic filters and anthropogenic propagule pressure primarily determine establishment success (Daly et al., 2023). Recent evidence demonstrates that native functional richness constrains invasions in stage-dependent contexts, with biotic resistance strength varying across invasion stages (Guo et al., 2024a). For example, during later invasion stages, *Solidago canadensis* utilizes plant height-mediated niche complementarity to limit light resource availability (Wang et al., 2018), a strategy that predominantly enhances invasion success. In this study, invasion levels are interpreted as proxies for progressive invasion stages – with heavy invasion reflecting later phases (e.g., naturalization-invasion), and light invasion aligning with earlier phases (e.g., introduction-naturalization). Thus, integrating invasion levels with multidimensional trait frameworks is therefore essential for unraveling how invasion trajectories reshape trait-mediated diversity-invasibility relationships.

This study systematically analyzes data from 290 herbaceous plots in the suburbs Jinhua, China, quantifying eight functional traits spanning four dimensions: Canopy structure, leaf traits, biomass allocation, and allometry for both invasive and native species. By analyzing native and invasive species’ traits differentially regulate community invasibility, we address three key questions: (1) How do community diversity and invasibility change along invasion levels? (2) Which native species traits confer resistance at different invasion levels? (3) What invasive traits drive invasion success as intensity increases?

## 2 Materials and methods

### 2.1 Study site and community census

**Fig. 1:**
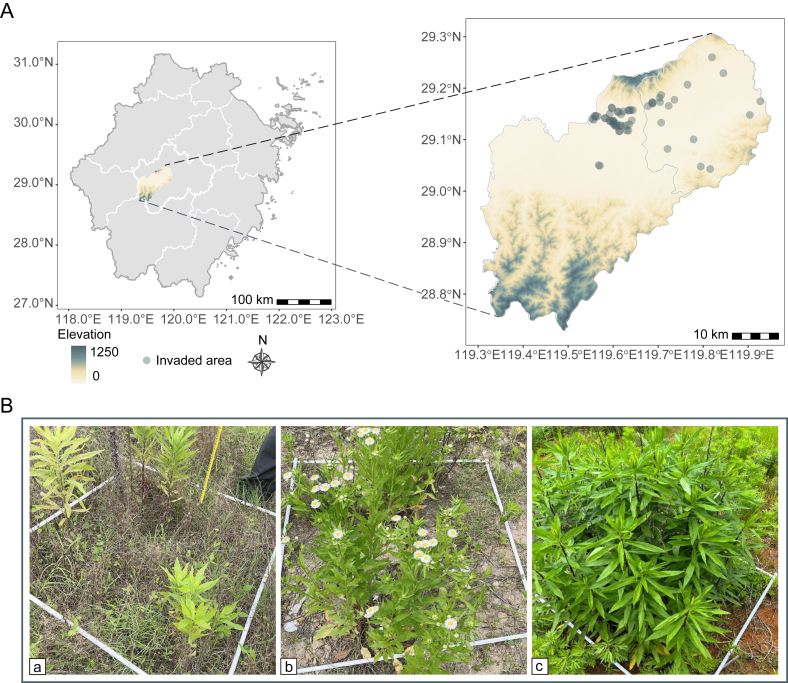
**Geographic location of invaded areas and classification of invasion levels. A,** Distribution of the 58 invaded areas in the Jinhua City, Zhejiang Province (the administrative map of Zhejiang Province is on the left, and Jindong District and Wucheng District in Jinhua City are on the right). **B,** Representative herbaceous plots illustrating the three invasion levels: (a) Light (RA ≤ 30%); (b) Moderate (30% < RA ≤ 70%); (c) Heavy (RA > 70%). Invasion levels were classified for each plot based on invasive species' relative abundance (RA).

The study was conducted in the suburbs of Jinhua City, Zhejiang Province, China (28.533–29.683°N, 119.233–120.775°E; Fig. 1). This region is characterized by a subtropical monsoon climate with a mean annual temperature of 17.5 °C, 1424 mm precipitation, and predominant red-yellow soils (Li et al., 2022a). We focused on suburban herbaceous habitats with high anthropogenic connectivity, which are characterized by frequent human-mediated species dispersal but minimal direct habitat modification (Delavaux et al., 2023).

From June to October 2023, we established 290 herbaceous plots (1 m × 1 m) across 58 contiguous invaded areas (each covering at least 666.67 m^2^), with a minimum spacing of 10 m between plots to ensure spatial independence. Plots were classified into three invasion levels based on invasive species’ relative abundance (RA; Wang et al., 2018): Light (RA ≤ 30%), moderate (30% < RA ≤ 70%), and heavy (RA > 70%) (Fig. 1 and Table S1). All individual plants within each plot were identified to species level, with the number of individuals per species recorded, and functional traits measured. In summary, the surveyed flora comprised 114 species belonging to 94 genera and 42 families, with Asteraceae and Poaceae representing the dominant families (Table S2).

### 2.2 Plant functional traits collection

In this study, eight continuous functional traits (Leaf area (LA), leaf thickness (LT), leaf dry weight (LDW), aboveground biomass (AB), underground biomass (UB), specific leaf area (SLA), root-shoot ratio (RSR), and mean plant height (MH); Table 1) were collected for analysis, representing multidimensional functions such as resource acquisition, biomass allocation (Godoy et al., 2012). For each species, three healthy and mature individuals (height ≥ 5 cm) were selected for trait measurement. For plots containing fewer than three individuals, supplementary samples were collected from adjacent areas following standardized protocols (Xu et al., 2015). Invasive species trait data were exclusively derived from field measurements. For species with insufficient individuals or undersized morphology preventing field measurements, missing trait values were supplemented from the TRY trait database (Kattge et al., 2020; https://www.try-db.org/; request ID 28680, 28681, 28683, 28684, 28685). This included leaf dry weight for 10 species, underground biomass for three species, aboveground biomass for two species, leaf thickness for 11 species, and leaf area for 10 species. The remaining trait data (94.17%) were obtained from standardized field measurements, prioritizing TRY entries that matched our sampling protocols.

**Table 1:** Variables and descriptions of measured traits.

Functional Trait	Abbreviated	Unit	Description
Leaf area	LA	cm^2^	Relative leaf area of three well-grown leaves in the plot
Leaf thickness	LT	cm	The thickness between the upper and lower epidermis of the leaf
Leaf dry weight	LDW	g	The mass of the leaves after all moisture has been removed
Aboveground biomass	AB	g	The leaves, stems, and branches of plants make up the biomass above the soil
Underground biomass	UB	g	The weight of all living plants below the surface, expressed as dry weight, including rhizomes, tubers and plate roots of all living roots
Specific leaf area	SLA	m^2^·kg^−1^	Ratio of leaf area to dry weight
Root-shoot ratio	RSR	–	The dry weight ratio of the underground to the aboveground parts of the plant
Mean plant height	MH	cm	The distance between the base of the main stem and the growing point

Functional traits related to competitive strategies, growth potential, photosynthetic efficiency, and resource partitioning were measured following standardized protocols for plant functional trait measurement (Cornelissen et al., 2003; Pérez-Harguindeguy et al., 2013). For all field-measured traits, we selected at least five intact, mature leaves per plot for species-level leaf trait measurements. Similarly, three whole individuals per species were excavated per plot for biomass-related trait quantification. Specifically, leaf area was determined by scanning fully expanded leaves using an Epson GT-15000 scanner and analyzed using ImageJ software (v.1.8.0). Leaf thickness was measured using a digital vernier caliper (±0.01 mm precision) by stacking three mature leaves and recording the mean value. Plant height was measured as the vertical distance from the main stem base to the apical meristem. For decumbent stems, maximum natural height was recorded without artificial extension. After separating aerial organs (leaves, stems) from subterranean tissues, all samples were oven-dried at 80 °C until constant mass were achieved and subsequently weighed to quantify aboveground biomass, underground biomass, and leaf dry mass. Specific leaf area (SLA, cm^2^·g^−1^) was calculated as the ratio of leaf area to leaf dry mass. Root-shoot ratio (RSR) was measured as underground biomass divided by aboveground biomass (Godoy et al., 2012).

### 2.3 Community invasibility index

Community invasibility was quantified using the Community Invasibility Index (CII):

$$\begin{array}{c} CII=1-\left({MaxP}_{i}-P_{i}\right) \end{array}$$

where $P_{i}$ represents the relative abundance of invasive species in the plot *i*, and ${MaxP}_{i}$ denotes the maximum relative abundance of invasive species across all invaded plots (Wang et al., 2020a).

### 2.4 Statistical analysis

#### 2.4.1 Changes in community diversity across invasion levels

To address the first research question, functional and taxonomic diversity metrics were calculated based on all species within the community across invasion levels (Dixon, 2003; Grenié and Gruson, 2023). Functional diversity was quantified via Functional Dispersion (FDis) and Rao’s Quadratic Entropy (Rao’s *Q*). FDis measures the distribution of species traits within multidimensional functional space by quantifying the dispersion of species abundances around their community-weighted centroid (Laliberté and Legendre, 2010). Rao’s *Q* integrates species abundance distribution and pairwise functional differences, reflecting trait dissimilarity contributions to overall community diversity (Rao, 1982; Fu et al., 2014). Taxonomic diversity was assessed using the Shannon–Wiener Index (*H*) and Pielou’s Evenness Index (*J*). Since Shapiro–Wilk tests indicated non-normality in data distributions (*p* < 0.05), inter-group comparisons among invasion levels were performed using non-parametric Wilcoxon rank-sum tests.

#### 2.4.2 Changes in community invasibility driven by species traits across invasion levels

To address the second and third research questions, community weighted means (CWMs) of native and invasive species traits were first calculated to assess functional dominance. The functional traits of invasive and native species were analyzed using CWMs across three invasion levels, with species-level traits weighted by their relative abundance within plots. Non-parametric Wilcoxon tests with Bonferroni correction were applied to assess trait differences between invasion levels, separately for invasive and native species groups. A dual analytical approach was adopted: 1) stratified generalized linear models (GLMs) examining invasion-level-specific relationships (stratified analysis), and 2) global GLMs incorporating all invasion levels (unstratified analysis). Gamma-distributed error structures with log-link functions were employed to accommodate right-skewed, non-negative response variables (Community Invasibility Indices, CII). Species richness and abundance were included as covariates to control for plot-scale effects. Model selection was conducted through all-subsets regression, with candidate models ranked by Akaike Information Criterion with a small sample correction (AICc). Best-fit models with fewer parameters were prioritized when equivalent explanatory power (ΔAICc ≤ 2) was observed (Burnham and Anderson, 2002; Wang et al., 2020b). To assess potential correlations among functional traits and validate the multidimensional trait framework (Díaz et al., 2016; Bergmann et al., 2020; Carmona et al., 2021), we performed principal component analysis (PCA). The PCA included all measured traits for both native and invasive species. Results are presented in Fig. S1.

All statistical analyses and visualizations were performed in R v.4.3.1 (http://www.r-project.org/). Community diversity metrics were calculated by employing the *vegan* package and the *FD* package. GLMs were fitted using the *lmerTest* package (Kuznetsova et al., 2017), and all-subsets regression was implemented via the *MuMIn* package (Barton, 2022). We performed PCA by using the *FactoMineR* package (Lê et al., 2008).

## 3 Results

### 3.1 Changes of community diversity and invasibility across invasion levels

**Fig. 2:**
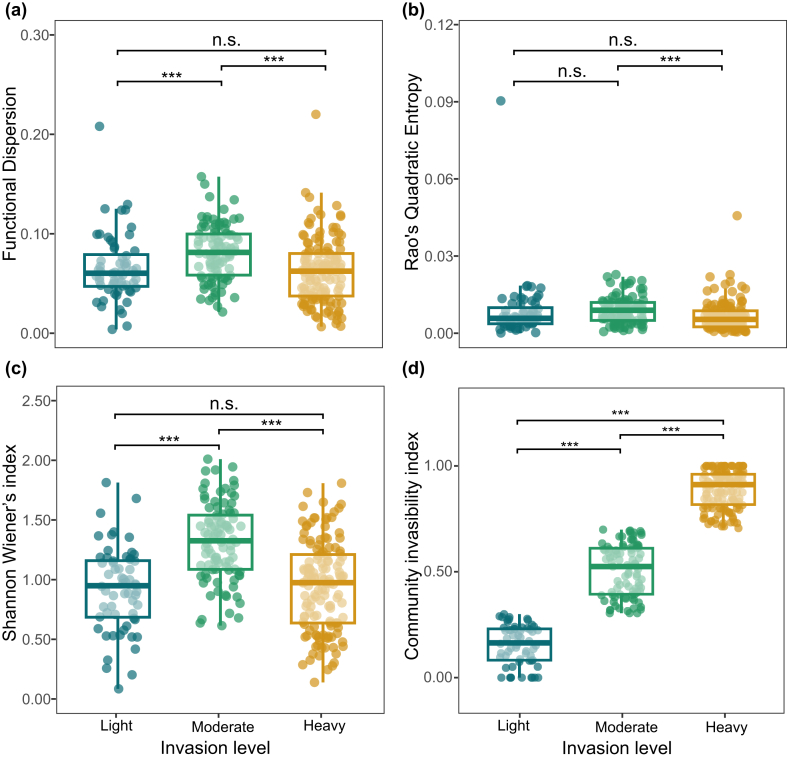
**Changes in community invasibility and diversity indices across invasion levels: (a) Functional Dispersion (FDis), (b) Rao’s Quadratic Entropy (Rao’s *Q*), (c) Shannon–Wiener’s index, (d) Community invasibility index (CII)**. Invasion levels were represented by light (light invasion), moderate (moderate invasion) and heavy (heavy invasion). Asterisks denote significance levels (∗*p* < 0.05; ∗∗*p* < 0.01; ∗∗∗*p* < 0.001).

Functional dispersion (FDis) and taxonomic diversity (Shannon and Pielou’s indices) followed hump-shaped patterns, peaking at moderate invasion levels (30% < RA ≤ 70%) and declining sharply under heavy invasion (RA > 70%) (Fig. 2a–c and Fig. S2). In contrast, Community invasibility increased significantly with invasion levels (Fig. 2d). The results of Spearman’s correlation for each indicator were shown in Fig. S3.

### 3.2 Effects of native functional traits on community invasibility across invasion levels

**Fig. 3:**
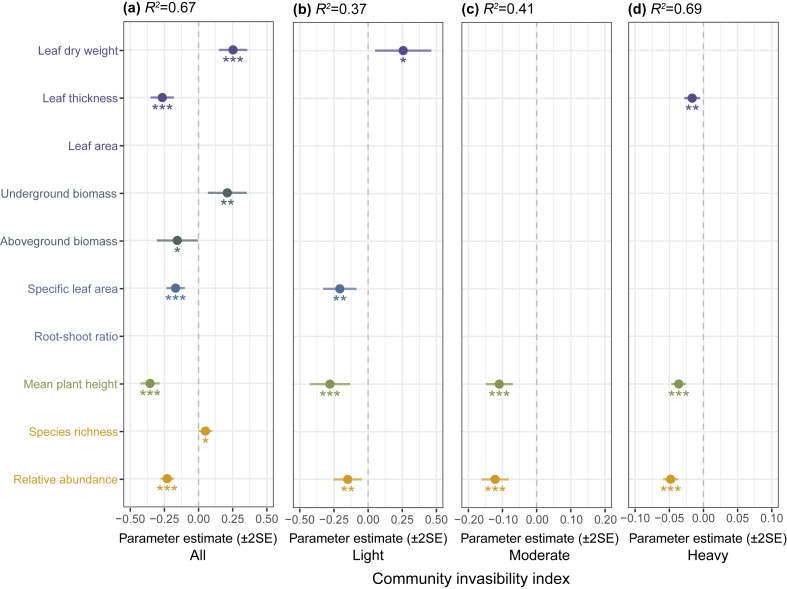
**Influence of native functional traits and diversity on community invasibility across invasion levels.** (a) All (that is unstratified analyses incorporate data from all plots), whereas stratified analyses categorize invasion level as light (light invasion, b), moderate (moderate invasion, c), heavy (heavy invasion, d). The values corresponding to the dots are the model fitting coefficients, with solid points denoting statistically significant relationships. Variables not included in the optimal model are empty values (∗*p* < 0.05; ∗∗*p* < 0.01; ∗∗∗*p* < 0.001).

Community invasibility was differentially regulated by native functional traits across invasion levels (Fig. 3). In unstratified analyses, leaf dry weight (LDW) significantly enhanced the invasibility. Biomass allocation exhibited contrasting effects: Underground biomass (UB) positively influencing invasibility whereas aboveground biomass (AB) suppressed it (Fig. 3a). When stratified by invasion level, LDW enhanced invasibility exclusively under light invasion (Fig. 3b). Leaf thickness (LT) and specific leaf area (SLA) exhibited opposing stage-dependent effects: LT reduced invasibility under heavy invasion (Fig. 3d), whereas SLA suppressed invasibility under light invasion (Fig. 3b). Notably, native species’ community-weighted mean (CWM) traits showed limited variation across invasion levels, with changes primarily constrained to leaf traits (Fig. S4). Canopy structure (mean plant height, MH) consistently reduced invasibility across all stages. Native abundance (Abu) was strongly negatively correlated with invasibility across all stages, whereas species richness (Ric) exhibited only weakly predictive power in unstratified analyses (Fig. 3a).

### 3.3 Trait-mediated invasibility of invasive species across invasion levels

**Fig. 4:**
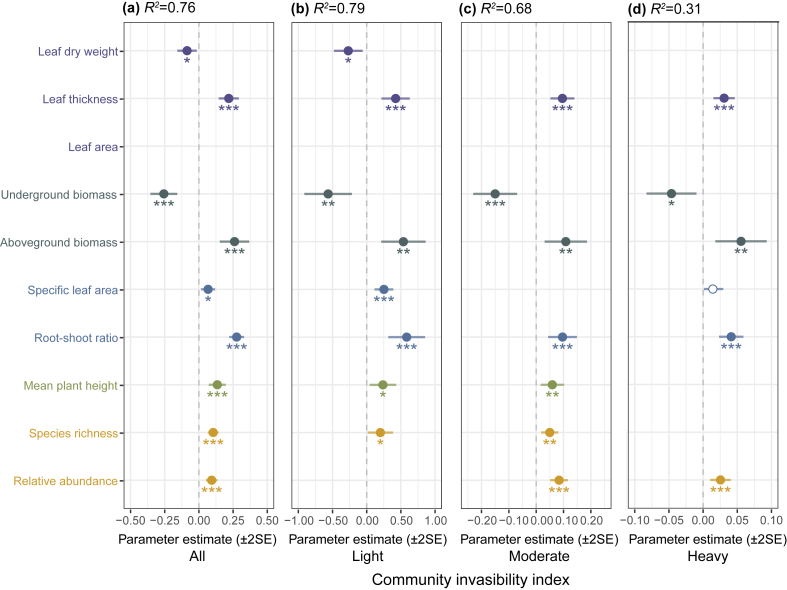
**Influence of invasive functional traits and diversity on community invasibility across invasion levels.** (a) All (that is unstratified analyses incorporate data from all plots), whereas stratified analyses categorize invasion level as light (light invasion, b), moderate (moderate invasion, c), heavy (heavy invasion, d). The values corresponding to the dots are the model fitting coefficients, with solid points denoting statistically significant relationships and hollow points indicating non-significant associations. Variables not included in the optimal model are empty values (∗*p* < 0.05; ∗∗*p* < 0.01; ∗∗∗*p* < 0.001).

Invasive species traits significantly influenced community invasibility (Fig. 4). LT consistently increased invasibility across all invasion stages, whereas LDW suppressed invasibility under light invasion (Fig. 4b). Biomass allocation strategies showed marked divergence: AB enhanced invasibility across all stages, while UB reduced it. Allometric traits (RSR and SLA) significantly promoted community invasibility, though SLA lost significance at moderate-to-heavy levels (Fig. 4). MH facilitated invasibility during light-to-moderate levels but showed no effect under heavy invasion level (Fig. 4b and c). Invader abundance (Abu) strongly amplified invasibility at moderate-to-heavy levels (Fig. 4c and d), whereas species richness (Ric) lost predictive power under heavy invasion (Fig. 4b and c). Notably, in unstratified analyses, only LDW and UB significantly suppressed invasibility, while all other variables mentioned above exhibited facilitative effects on invasibility (Fig. 4a). Despite varying invasion intensities, invasive species’ CWM traits remained stable across gradients (Fig. S5).

## 4 Discussion

### 4.1 Invasion level-driven changes in community invasibility and biodiversity

The hump-shaped patterns observed in functional and taxonomic diversity, peaking at moderate invasion levels (Fig. 2a–c), consistent with the intermediate disturbance hypothesis (Connell, 1978), where moderate disturbances optimize biodiversity by balancing competitive exclusion and resource availability. In invaded communities, this likely reflects invasive species-induced microhabitat heterogeneity that facilitates native-invasive coexistence (Cavieres, 2021). This mechanism resonates with Elton’s empty niche hypothesis (Elton, 1958), where initial invasions occupy unoccupied functional niches, thereby explaining the observed functional dispersion (FDis) enhancement. In addition, the increased FDis under light-to-moderate invasion levels indicates strengthened niche complementarity among species (Li et al., 2022b). However, this diversity peak represents a metastable equilibrium, as progressive niche saturation precedes eventual collapse under heavy invasion.

The decline of functional and taxonomic diversity under heavy invasion (Fig. 2a–c) reflects competitive exclusion dynamics. At this stage (i.e., the advanced invasion phase), resource limitation intensifies competition between native and invasive species (Wang et al., 2018), consistent with the biotic interaction-dominated dynamics characteristic of later invasion stages (Richardson and Pyšek, 2012). This transition corresponds to the naturalization-invasion threshold, where invaders achieve demographic dominance (Guo et al., 2024a), as evidenced by peak diversity metrics observed at moderate invasion levels (Fig. 2a–c). Such dominance likely drives native species displacement and niche loss, primarily resulting in decreased taxonomic diversity. Moreover, the proximity of invasive populations to niche optima enhances their competitive superiority through higher reproductive rates (Liu et al., 2025), ultimately causing functional homogenization as native species are supplanted by those of invasive species. Besides, the relatively minor changes in functional diversity (Fig. 2a and b) also support our results that the CWMs of both native and invasive species showed limited overall variation across different invasion levels (Figs. S4 and S5). These findings collectively highlight interspecific competition and niche competition as key drivers of biodiversity erosion in heavily invaded communities.

Community invasibility increased with invasion levels (Fig. 2d) is consistent with empirical evidence demonstrating that progressive invasive dominance erodes biotic resistance. For instance, Cavieres (2021) showed that native-invasive interactions can fundamentally alter community dynamics through facilitative mechanisms, paradoxically increasing invasion success. Our findings corroborate the “invasional meltdown” hypothesis (Simberloff and Von Holle, 1999), which initial invasions lower competitive barriers for subsequent colonizers. The accelerated invasibility under heavy invasion aligns with the concept of cumulative stress weakening community resistance.

### 4.2 Stage-dependent modulation of community invasibility by native functional traits

Our findings reveal that native functional traits regulate community invasibility through invasion stage-specific mechanisms (Fig. 3). While leaf dry weight (LDW) enhanced invasibility in unstratified analyses, its effects were restricted to light invasion stages (Fig. 3a and b), likely reflecting its dual role as a proxy for resource allocation strategies. LDW links rapid leaf turnover and high photosynthetic efficiency (Liao et al., 2021), reflecting a conservative resource strategy that improves environmental adaptation but reduces invasion resistance through resource trade-offs. This negative correlation with community invasibility that prioritizing conservative traits may drive biological invasions via ecological compromises. Conversely, under heavy invasion, leaf thickness (LT) emerged as a critical resistance trait, suppressing invasibility (Fig. 3d). This aligns with evidence that thicker leaves enhance physical defense against herbivory and microenvironmental stress (Niinemets et al., 2003), establishing biotic filters that limit invader establishment (Zhao et al., 2024; Wang et al., 2025). These stage-dependent dynamics reflect that leaf economic traits mediate invasibility through trait-environment feedbacks. Conservative strategies (e.g., higher LDW) become ineffective under intensified competition, whereas structural investments (e.g., LT) assume greater prominence in late-stage resistance. Besides, the community-weighted mean (CWM) of leaf thickness significantly increased under heavy invasion (Fig. S4), which also directly evidencing native communities’ structural reinforcement in response to invader dominance.

Besides, biomass allocation exhibited divergent pathways, as evidenced by unstratified analysis (Fig. 3a). Underground biomass (UB) significantly promoted invasibility, likely by enriching soil nutrients for invaders (Cavieres, 2021), whereas aboveground biomass (AB) suppressed it through resource competition (Wang et al., 2021). This suggests that native resistance primarily operates through aboveground trait-mediated interference with invader growth and reproduction (Wang et al., 2014). Allometric traits like SLA further reinforced stage dependency. The inhibition effect of SLA on invasibility under light invasion (Fig. 3b) aligns with growth-defense tradeoffs, wherein low-SLA natives prioritize structural resilience over rapid growth (Cornelissen et al., 2003). This mechanism is particularly critical during early invasion stage, where native-invasive competition is most pronounced. Notably, SLA significantly inhibited invasibility in unstratified analyses (Fig. 3a), highlighting its broad influence, which is mechanistically linked to species-specific photosynthetic rates (Liao et al., 2021). However, this relationship masked as invasion intensity varies across stages.

In contrast, canopy structure traits (e.g., mean plant height, MH) consistently suppressed invasibility across all invasion stages (Fig. 3). Taller native canopies inhibit community invasibility by limiting light availability – a critical mechanism for excluding shade-intolerant invaders (Wang et al., 2021). This result corroborates previous findings that taller native species outcompete invaders through resource limitation (Gross et al., 2007; Thomson et al., 2011). Moreover, the vertical stratification of native canopies establishes physical barriers to invasive plant establishment (Wang et al., 2021). Biodiversity indices exhibited contrasting effects. Native abundance (Abu) strongly inhibited invasibility across all invasion stages (Fig. 3a–d), supporting Elton’s biotic resistance hypothesis (Elton, 1958). In contrast, species richness (Ric) showed weak positive correlations only in unstratified models (Fig. 3a), consistent with the finding that high native abundance reduces invader success by monopolizing both abiotic and biotic resources (Cubino et al., 2022; Guo et al., 2024a). This underscores that dominance of native functional traits, rather than taxonomic diversity alone, drives invasion resistance.

### 4.3 Invasion stage dependency of invader trait-mediated community invasibility

Our results demonstrate that invasive species’ functional traits regulate community invasibility through mechanisms contingent on invasion stages. Leaf traits exhibited divergent roles. LT consistently enhanced invasibility across all invasion stages (Fig. 4a–d), likely by reducing herbivory pressure and enhancing stress tolerance (Zhao et al., 2024). However, LDW suppressed invasibility under light invasion and in unstratified models (Fig. 4a and b), reflecting trade-offs between photosynthetic efficiency and competitive costs (Liao et al., 2021). Hence, the effect of LDW is stage-dependent, with its effects being most pronounced during early stages when resource investment is critical. These divergent patterns align with the inherent superiority hypothesis (Lowry et al., 2013), wherein thicker leaves confer broad adaptive advantages. Invasive species overcomes early colonization challenges by relying on such leaf trait advantages.

Biomass allocation further revealed adaptive strategies. AB strongly promoted invasibility across all invasion stages (Fig. 4), while UB consistently suppressed it. This asymmetric pattern reflects invasive plants’ prioritization of aboveground resource competition, which is critical for securing light and space in new environments (Wang et al., 2014). Although invasive species may enhance soil nutrient availability through mycorrhizal symbiosis (Negesse et al., 2025), excessive underground allocation could compromise their capacity to dominate aboveground resource competition. Such allocation patterns align with our present study that invaders in resource-rich habitats optimize aboveground biomass distribution (e.g., reduced RSR) to maximize resource capture (Funk, 2008). This also indirectly supports the hypothesis that invasive species favor aboveground dispersal strategies (Guerra-García et al., 2018; Rathee et al., 2021). Allometric traits (RSR, SLA) promoted invasibility in unstratified analyses (Fig. 4a), but only RSR retained significance under moderate and heavy invasion (Fig. 4c and d). Root dominance in soil enhances competition and accelerates the process of invasion (Cui et al., 2023; Negesse et al., 2025). This strategy likely synergizes with high SLA, which correlates with rapid biomass accumulation and early-stage dominance (Cui et al., 2023).

Canopy structure traits (e.g., MH) facilitated invasibility during light-to-moderate invasion and in unstratified model (Fig. 4a–c). This highlights the importance of plant height in early resource competition, directly reflects competitive ability (Li et al., 2022b; Stotz et al., 2025). Biodiversity drivers exhibited stage-dependent shifts, Ric promoted invasibility only during early invasion stage (Fig. 4b and c), whereas Abu dominated later stages (Fig. 4c and d). This likely reflects a transition from diversity-driven to dominance-driven invasion mechanisms. In the unstratified analysis, both Ric and Abu significantly promoted invasibility (Fig. 4a), indicating that the diversity of invasive species enhances invasion success rates in local communities (Cavieres, 2021). Collectively, these findings emphasize that invasion success hinges on the spatiotemporal coordination of functional traits, balancing immediate colonization demands with long-term resource monopolization.

## 5 Conclusions

Our study demonstrates that invasion dynamics in subtropical urban ecosystems are contingent on the stage-dependent coordination of multidimensional functional traits. The observed hump-shaped diversity-invasibility relationship consistent with the intermediate disturbance hypothesis while extending this framework by revealing how invasive species exploit niche complementarity during moderate invasion phases (30% < RA ≤ 70%) before driving competitive exclusion under heavy invasion (RA > 70%). Native communities employ dual defense strategies: Structural traits (e.g., canopy height) confer cross-stage resistance through persistent resource competition, while leaf traits (e.g., thickness) assume greater functional prominence under heavy invasion stage by enhancing stress tolerance and biotic resistance. These findings indicate that native abundance, rather than taxonomic richness, serve as the primary driver of biotic resistance – a pattern consistent with Elton’s hypothesis. This highlights the pivotal role of functional dominance over taxonomic diversity in mediating invasion outcomes. In contrast, invasive species exhibit adaptive plasticity, transitioning from rapid colonization traits (height advantage) to stress-tolerant strategies (thick leaves, root-shoot optimization). Importantly, invader trait coordination supersedes diversity effects at heavy stages, aligning with the inherent superiority hypothesis wherein coordinated traits confer broad competitive advantages.

From a management perspective, urban ecosystem management should prioritize structural preservation (e.g., maintaining native canopy height) to mitigate early-stage invasions, combined with targeted augmentation of stress-tolerant traits (e.g., enhancing leaf thickness) in areas experiencing heavily invasion. While this study was confined to herbaceous communities in subtropical urban ecosystems, future research should validate these patterns across broader ecological contexts (e.g., forests, wetlands) and functional groups (e.g., woody perennials, annual grasses). Furthermore, integrating longitudinal analyses of trait-environment feedbacks is essential to elucidate causal mechanisms underlying invasion trajectories and their ecosystem-level consequences.

## CRediT authorship contribution statement

Chengwei Li: Writing – review & editing, Writing – original draft, Methodology, Formal analysis, Visualization, Conceptualization. Qi Wu: Writing – original draft, Validation, Methodology, Visualization, Cheng Du: Writing – review & editing, Investigation, Data curation. Laihong Gu: Writing – review & editing, Investigation, Data curation. Xingchen Wang: Writing – review & editing, Investigation, Data curation. Jiajie Xie: Writing – review & editing, Investigation, Data curation. Jiayi Wang: Writing – review & editing, Investigation, Data curation. Jianhua Chen: Writing – review & editing, Data curation, Supervision, Funding acquisition, Conceptualization. Yunquan Wang: Writing – review & editing, Validation, Methodology, Investigation, Data curation, Conceptualization.

## Declaration of competing interest

The authors declare that they have no known competing financial interests or personal relationships that could have appeared to influence the work reported in this paper.
