# Supplementary material for: Stage-dependent shifts in native and invasive traits mediate community invasibility in subtropical urban ecosystems
Source: Plant Divers. 2025 Jun 24;48(2):389–98. doi: 10.1016/j.pld.2025.06.007 (PMC13071453; doi:10.1016/j.pld.2025.06.007)

# Appendix

**Table S1** Information on the composition of plots across invasion levels.

| **Invasion Level** | **Number of Plots** | **Invasive Species Richness** | | | **Total Species Richness** | | | **Invasive Species Abundance** | | | **Total Species Abundance** | | |
| --- | --- | --- | --- | --- | --- | --- | --- | --- | --- | --- | --- | --- | --- |
|  |  | **Range** | **Mean** | **SD** | **Range** | **Mean** | **SD** | **Range** | **Mean** | **SD** | **Range** | **Mean** | **SD** |
| Light | 60 | 0-6 | 2.13 | 1.33 | 2-9 | 5.17 | 1.84 | 0-41 | 11.10 | 9.89 | 9-496 | 71.56 | 68.97 |
| Moderate | 92 | 1-6 | 3.11 | 1.33 | 2-10 | 6.17 | 1.86 | 5-138 | 29.42 | 20.81 | 9-228 | 57.20 | 34.96 |
| Heavy | 138 | 1-6 | 3.30 | 1.24 | 1-10 | 4.85 | 1.73 | 5-268 | 44.30 | 34.22 | 7-289 | 50.02 | 36.99 |

**Table S2** List of native and invasive species surveyed in the study area.

| **Species** | **Family** | **Species Categorization** |
| --- | --- | --- |
| *Alternanthera philoxeroides* | Amaranthaceae | Invasive species |
| *Amaranthus retroflexus* | Amaranthaceae | Invasive species |
| *Phytolacca america* | Phytolaccaceae | Invasive species |
| *Ipomoea purpurea* | Convolvulaceae | Invasive species |
| *Ipomoea triloba* | Convolvulaceae | Invasive species |
| *Solidago cadensis* | Asteraceae | Invasive species |
| *Symphyotrichum subulatum* | Asteraceae | Invasive species |
| *Erigeron annuus* | Asteraceae | Invasive species |
| *Erigeron canadensis* | Asteraceae | Invasive species |
| *Erigeron sumatrensis* | Asteraceae | Invasive species |
| *Bidens frondosa* | Asteraceae | Invasive species |
| *Bidens pilosa* | Asteraceae | Invasive species |
| *Lepidium virginicum* | Brassicaceae | Invasive species |
| *Trifolium repens* | Fabaceae | Invasive species |
| *Geranium carolinianum* | Geraniaceae | Invasive species |
| *Daucus carota* | Apiaceae | Invasive species |
| *Veronica persica* | Scrophulariaceae | Invasive species |
| *Conyza boriensis* | Asteraceae | Invasive species |
| *Euphorbia hypericifolia* | Euphorbiaceae | Invasive species |
| *Plantago virginica* | Plantaginaceae | Invasive species |
| *Cyclospermum leptophyllum* | Apiaceae | Invasive species |
| *Cerastium glomeratum* | Caryophyllaceae | Invasive species |
| *Cosmos bipinta* | Asteraceae | Invasive species |
| *Euphorbia maculata* | Euphorbiaceae | Invasive species |
| *Lolium perenne* | Poaceae | Invasive species |
| *Medicago sativa* | Fabaceae | Invasive species |
| *Melilotus officinalis* | Fabaceae | Invasive species |
| *Oxalis corymbosa* | Geraniaceae | Invasive species |
| *Sida acuta* | Malvaceae | Invasive species |
| *Sonchus asper* | Asteraceae | Invasive species |
| *Sonchus oleraceus* | Asteraceae | Invasive species |
| *Oenothera speciosa* | Onagraceae | Invasive species |
| *Verbena bonariensis* | Verbenaceae | Invasive species |
| *Triodanis perfoliate* subsp. *biflora* | Campanulaceae | Invasive species |
| *Xanthium strumarium* | Asteraceae | Invasive species |
| *Lactuca serriola* | Asteraceae | Invasive species |
| *Equisetum ramosissimum* | Equisetaceae | Native species |
| *Lygodium japonicum* | Lygodiaceae | Native species |
| *Woodwardia japonica* | Blechnaceae | Native species |
| *Celtis sinensis* | Ulmaceae | Native species |
| *Broussonetia papyrifera* | Moraceae | Native species |
| *Humulus scandens* | Moraceae | Native species |
| *Boehmeria nivea* | Urticaceae | Native species |
| *Fagopyrum dibotrys* | Polygonaceae | Native species |
| *Persicaria criopolitana* | Polygonaceae | Native species |
| *Persicaria lapathifolia var. salicifolia* | Polygonaceae | Native species |
| *Persicaria hydropiper* | Polygonaceae | Native species |
| *Persicaria perfoliata* | Polygonaceae | Native species |
| *Rumex acetosa* | Polygonaceae | Native species |
| *Chenopodium album* | Chenopodiaceae | Native species |
| *Portulaca oleracea* | Portulacaceae | Native species |
| *Ranunculus japonicus* | Ranunculaceae | Native species |
| *Rorippa globosa* | Brassicaceae | Native species |
| *Rubus hirsutus* | Rosaceae | Native species |
| *Rubus parvifolius* | Rosaceae | Native species |
| *Potentilla kleiniana* | Rosaceae | Native species |
| *Duchesnea indica* | Rosaceae | Native species |
| *Rosa bracteata* | Rosaceae | Native species |
| *Agrimonia pilosa* | Rosaceae | Native species |
| *Aeschynomene indica* | Fabaceae | Native species |
| *Vicia hirsuta* | Fabaceae | Native species |
| *Vicia sativa* | Fabaceae | Native species |
| *Medicago lupuli* | Fabaceae | Native species |
| *Kummerowia striata* | Fabaceae | Native species |
| *Pueraria montana* var. *lobata* | Fabaceae | Native species |
| *Glycine soja* | Fabaceae | Native species |
| *Oxalis corniculata* | Oxalidaceae | Native species |
| *Phyllanthus urinaria* | Euphorbiaceae | Native species |
| *Triadica sebifera* | Euphorbiaceae | Native species |
| *Acalypha australis* | Euphorbiaceae | Native species |
| *Rhus chinensis* | Anacardiaceae | Native species |
| *Vitis pseudoreticulata* | Vitaceae | Native species |
| *Causonis japonica* | Vitaceae | Native species |
| *Hydrocotyle vulgaris* | Apiaceae | Native species |
| *Torilis scabra* | Apiaceae | Native species |
| *Lysimachia candida* | Primulaceae | Native species |
| *Cynanchum rostellatum* | Asclepiadaceae | Native species |
| *Caryopteris inca* | Verbenaceae | Native species |
| *Leonurus japonicus* | Lamiaceae | Native species |
| *Clinopodium chinense* | Lamiaceae | Native species |
| *Solanum nigrum* | Solanaceae | Native species |
| *Mazus pumilus var. pumilus* | Scrophulariaceae | Native species |
| *Campsis grandiflora* | Bignoniaceae | Native species |
| *Paederia foetida* | Rubiaceae | Native species |
| *Galium spurium* | Rubiaceae | Native species |
| *Pseudognaphalium affine* | Asteraceae | Native species |
| *Artemisia annua* | Asteraceae | Native species |
| *Artemisia argyi* | Asteraceae | Native species |
| *Artemisia lavandulifolia* | Asteraceae | Native species |
| *Hemisteptia lyrata* | Asteraceae | Native species |
| *Centaurea cyanus* | Asteraceae | Native species |
| *Lactuca indica* | Asteraceae | Native species |
| *Ixeris polycephala* | Asteraceae | Native species |
| *Beckmannia syzigachne* | Poaceae | Native species |
| *Bromus japonicus* | Poaceae | Native species |
| *Elymus kamoji* | Poaceae | Native species |
| *Eleusine indica* | Poaceae | Native species |
| *Cynodon dactylon* | Poaceae | Native species |
| *Polypogon fugax* | Poaceae | Native species |
| *Alopecurus aequalis* | Poaceae | Native species |
| *Echinochloa colona* | Poaceae | Native species |
| *Echinochloa crus-galli* | Poaceae | Native species |
| *Paspalum thunbergii* | Poaceae | Native species |
| *Digitaria sanguinalis* | Poaceae | Native species |
| *Setaria faberii* | Poaceae | Native species |
| *Setaria viridis* | Poaceae | Native species |
| *Setaria pumila* | Poaceae | Native species |
| *Miscanthus floridulus* | Poaceae | Native species |
| *Imperata cylindrica* | Poaceae | Native species |
| *Cyperus iria* | Poaceae | Native species |
| *Kyllinga polyphylla* | Cyperaceae | Native species |
| *Reineckea carnea* | Asparagaceae | Native species |
| *Smilax china* | Liliaceae | Native species |
| *Allium macrostemon* | Liliaceae | Native species |

**Fig. S1** Principal component analysis (PCA) of functional traits for native and invasive species. Biplot of the first two principal components (PC1: 33.40% variance; PC2: 17.60%). Arrows indicate trait loadings, with direction and length reflecting contribution to each axis. Distribution of species scores, colored by species type. Ellipses represent 95% confidence intervals.

**
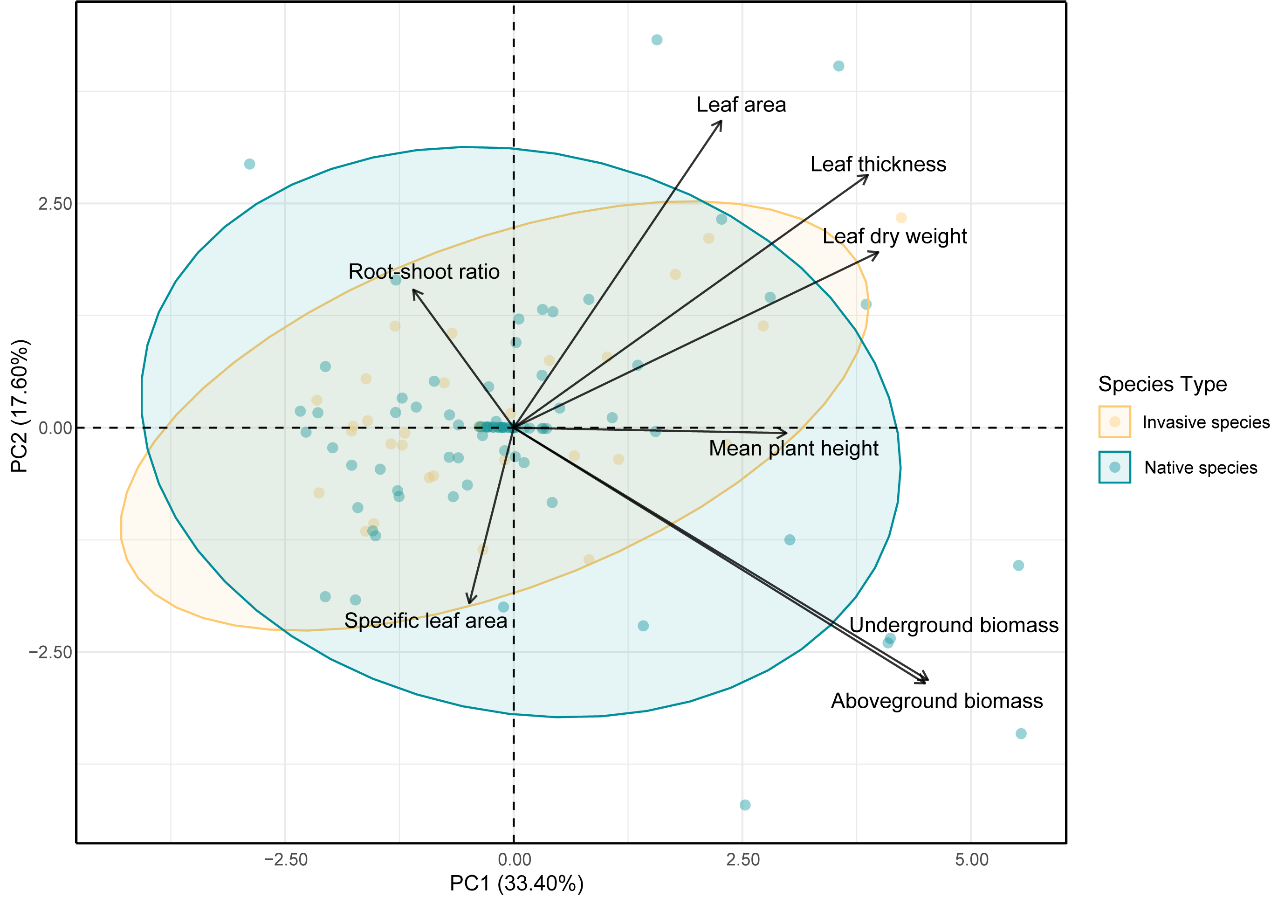
**

**Fig. S2** Changes in Pielou’s index across invasion levels. Invasion levels were represented by light (light invasion), moderate (moderate invasion) and heavy (heavy invasion). (*p < 0.05; **p < 0.01; ***p < 0.001).


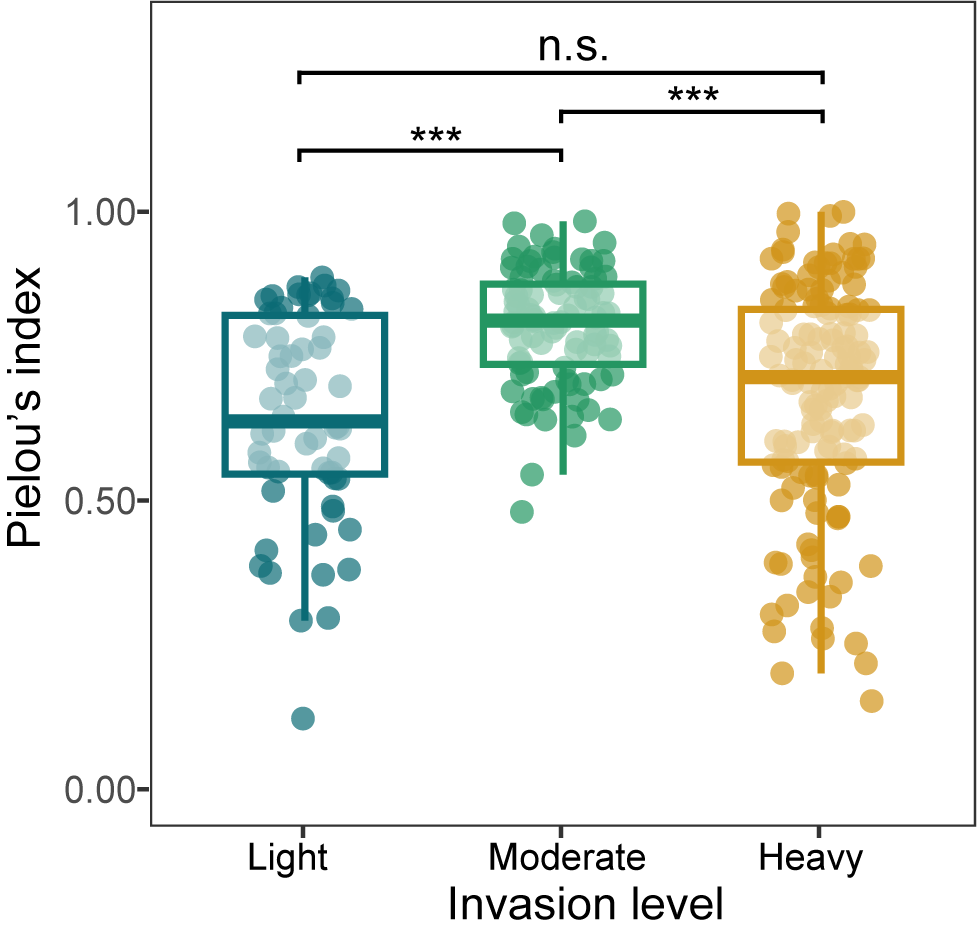
**Fig. S3** Correlation between study metrics and CII across invasion levels. (a) The correlation about native species; (b) The correlation about invasive species (*p < 0.05; **p < 0.01; ***p < 0.001).
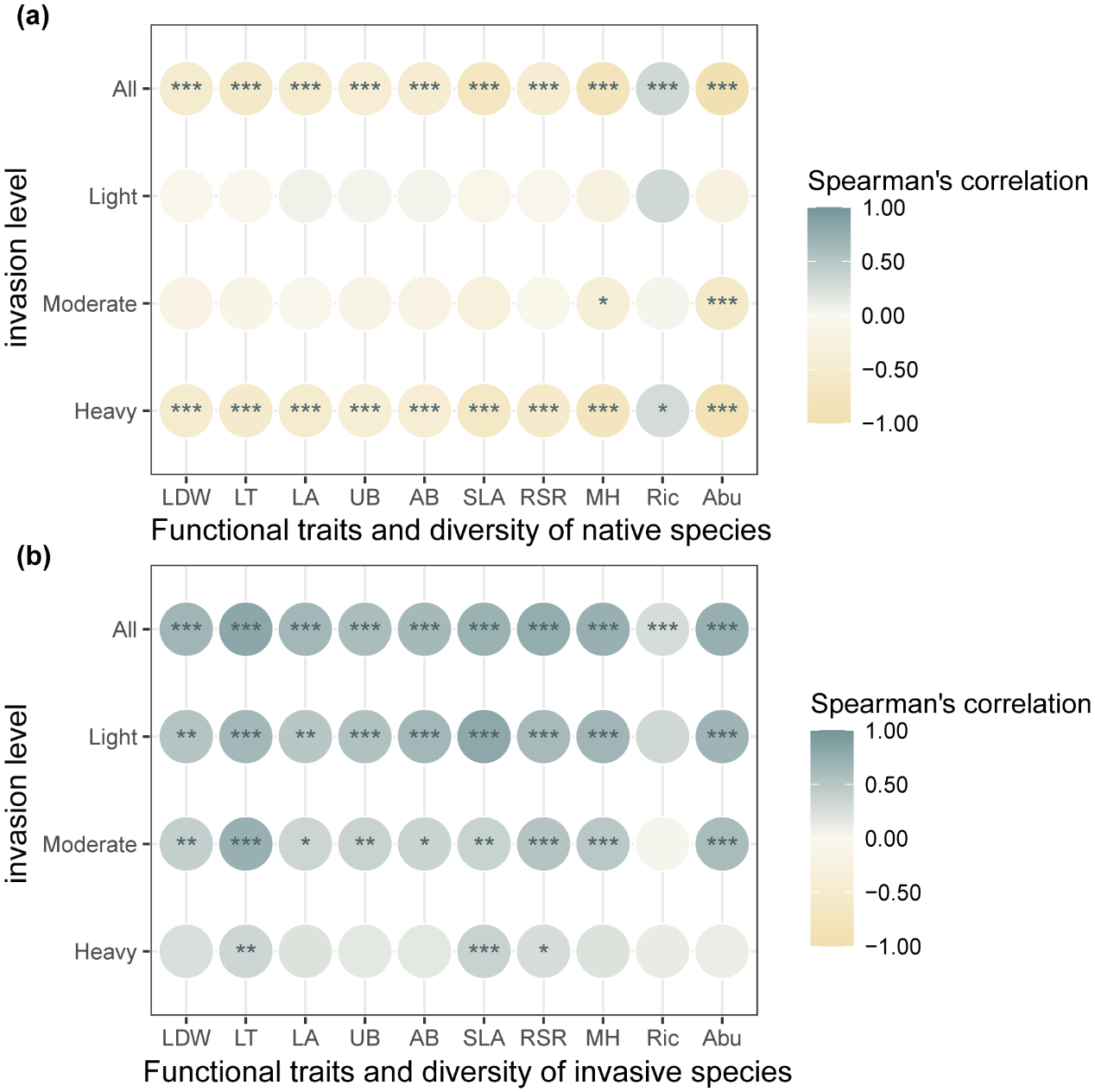


**Fig. S4** Community weighted mean (CWM) of functional traits in native species across invasion levels (*p < 0.05; **p < 0.01).


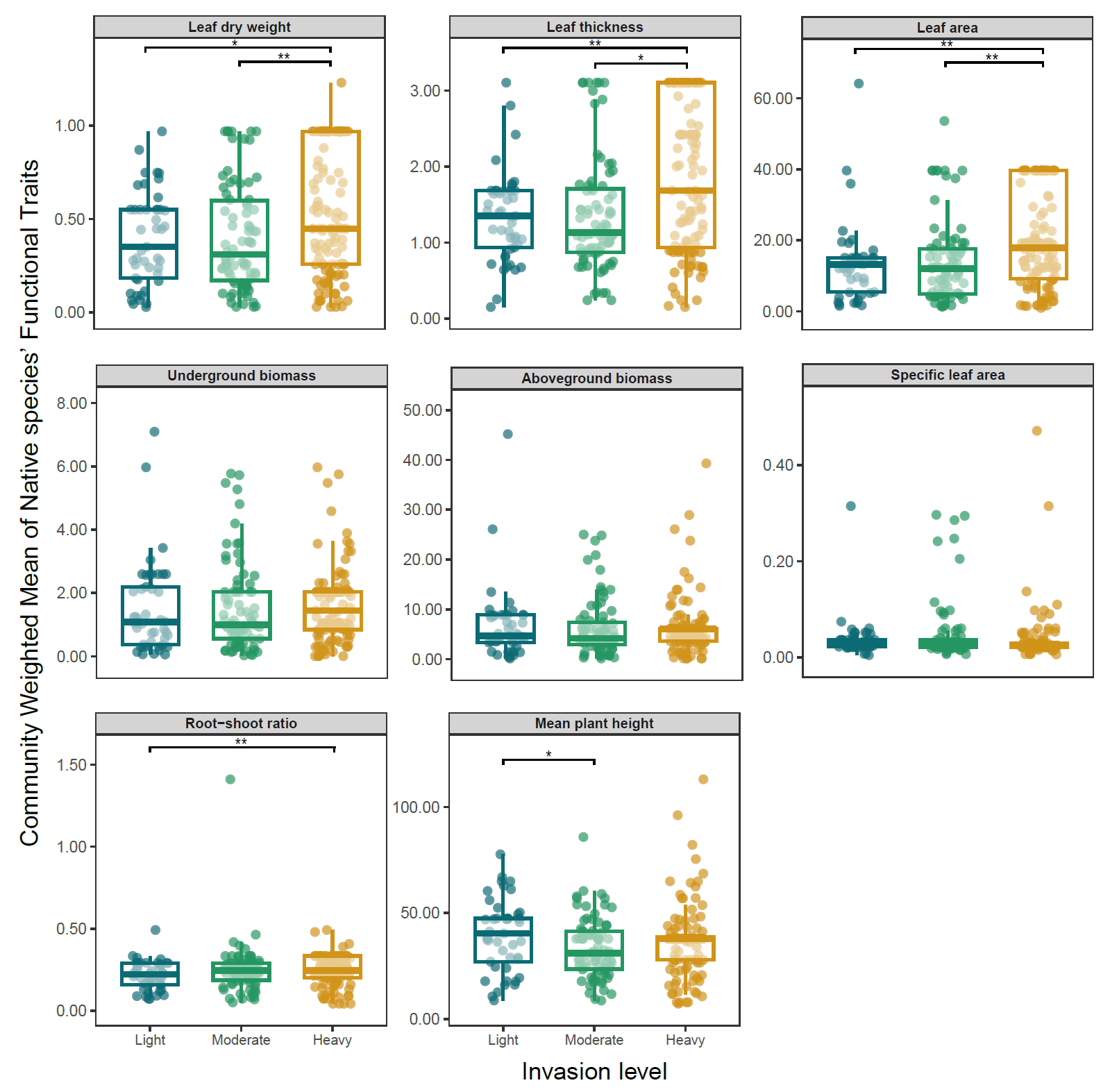


**Fig. S5** Community weighted mean (CWM) of functional traits in invasive species across invasion levels (*p < 0.05).


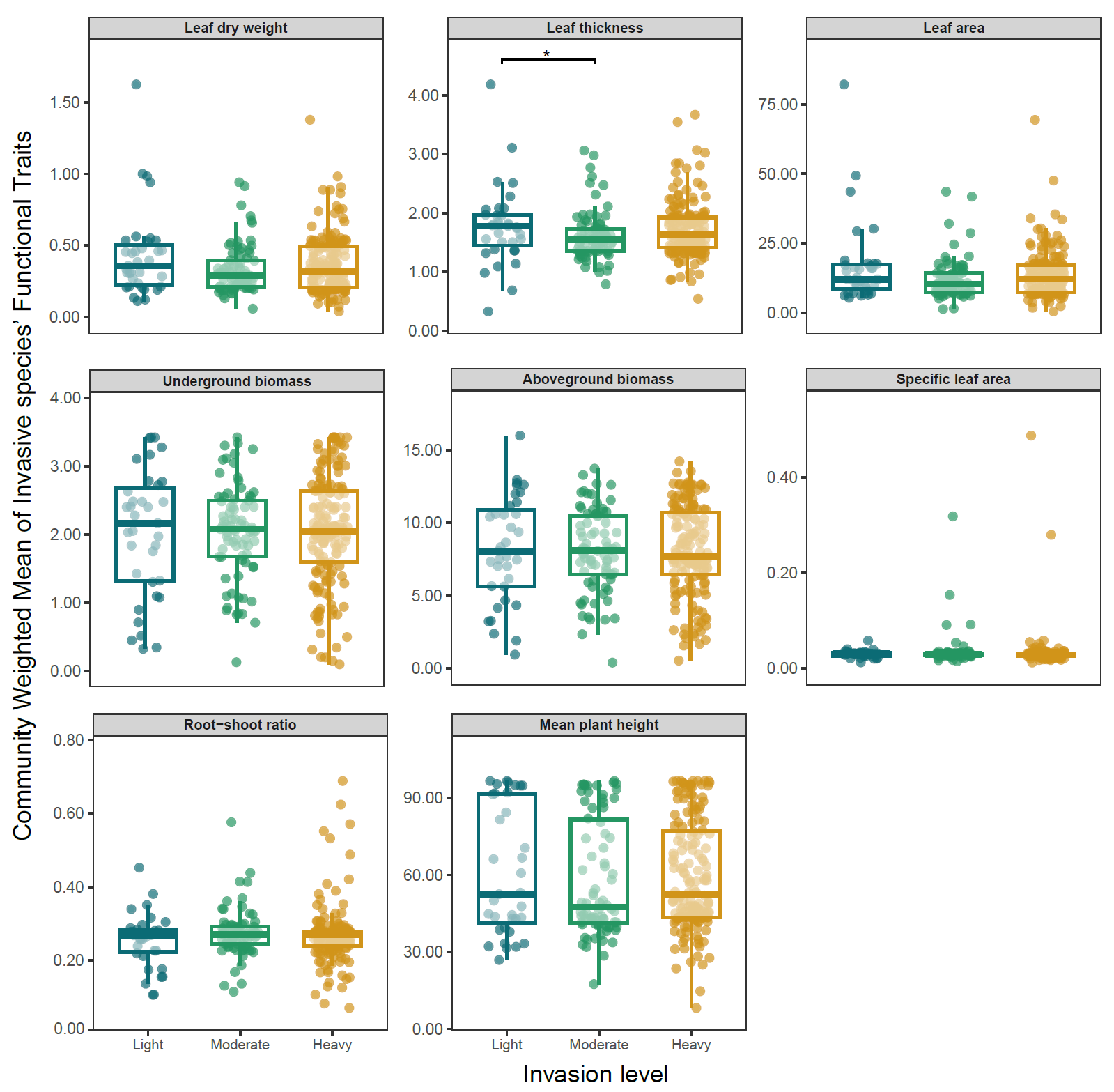

Supplement: Multimedia component 1 [file mmc1.docx]
